# Tgif1 and Tgif2 Regulate Axial Patterning in Mouse

**DOI:** 10.1371/journal.pone.0155837

**Published:** 2016-05-17

**Authors:** Tiffany A. Melhuish, Kenichiro Taniguchi, David Wotton

**Affiliations:** Department of Biochemistry and Molecular Genetics, and Center for Cell Signaling, University of Virginia, Charlottesville, United States of America; Columbia University, UNITED STATES

## Abstract

Tgif1 and Tgif2 are transcriptional repressors that inhibit the transcriptional response to transforming growth factor β signaling, and can repress gene expression by direct binding to DNA. Loss of function mutations in *TGIF1* are associated with holoprosencephaly (HPE) in humans. In mice, embryos lacking both Tgif1 and Tgif2 fail to complete gastrulation, and conditional double null embryos that survive past gastrulation have HPE and do not survive past mid-gestation. Here we show that in mice of a relatively pure C57BL/6 strain background, loss of Tgif1 alone results in defective axial patterning and altered expression of *Hoxc6*. The primary defects in *Tgif1* null embryos are the presence of extra ribs on the C7 vertebra, consistent with a posterior transformation phenotype. In addition we observed defective cervical vertebrae, primarily C1-C5, in both adult mice and embryos that lacked Tgif1. The combination of *Tgif1* and *Tgif2* mutations increases the severity and penetrance of the posterior transformation phenotype, without altering the type of defects seen. Similarly, exposure of *Tgif1* mutant embryos to retinoic acid at E8.5 increased the severity and penetrance of the *Tgif1* phenotype. This suggests that Tgif1 and Tgif2 regulate axial patterning and that reduced TGIF function sensitizes embryos to the effects of retinoic acid.

## Introduction

Tgif1 (thymine-guanine interacting factor) was first identified as a protein which binds a retinoid response element (RXRE) from the rat cellular retinol binding protein II (CRBPII) gene [1]. Tgif1 is a homeodomain protein of the TALE (three amino acid loop extension) superfamily, which have a three amino acid insertion between helices one and two of the homeodomain [1, 2]. Tgif family members are characterized by a highly conserved homeodomain and an approximately 20 amino acid carboxyl-terminal extension [3]. Outside this the similarity is more limited, although both Tgif1 and Tgif2 share a highly conserved transcriptional repression domain near their carboxyl-termini [3–5]. Tgif1 interacts directly with the mSin3 corepressor complex via the carboxyl-terminal domain and recruits histone deacetylases [6–8]. A short amino acid motif (PLDLS) within the Tgif1 amino-terminal repression domain interacts with the general transcriptional corepressor CtBP (Carboxyl-terminus Binding Protein) [9], which is part of a large corepressor complex [10, 11]. Tgif2 shares a high degree of sequence similarity to Tgif1, particularly over the homeodomain and carboxyl-terminal repression domain but lacks the amino-terminal CtBP-interaction motif [5, 12].

Tgif1 and Tgif2 are best characterized as regulators of Transforming Growth Factor ß (TGFß) responsive gene expression that act as Smad transcriptional corepressors [13, 14]. In response to binding of a TGFß family ligand to its receptors, the receptor complex phosphorylates and activates specific receptor Smad (R-Smad) proteins: Smad2 or Smad3 in the case of TGFß Nodal and Activin [15–17]. Activated R-Smads complex with the co-Smad, Smad4, translocate to the nucleus and activate target gene expression via interactions with general coactivators, such as p300/CBP [18]. The presence of specific Smad corepressors, such as TGIF1, limits the transcriptional response by competing with coactivators and by recruiting corepressor complexes [13, 14]. In addition to repressing TGFß-activated gene expression, Tgif1 interacts with the retinoid X receptor, and potentially other nuclear receptors, and recruits transcriptional corepressors [19, 20]. As well as being recruited to DNA indirectly, Tgifs can bind directly to DNA and repress gene expression [6, 21, 22]. Recent ChIP-seq analysis in mouse ES cells has identified a potentially large number of Tgif1 binding sites across the genome, many of which contain consensus Tgif binding sites, suggesting that this might be a major way that Tgifs regulate gene expression [23].

Mutations in the human *TGIF1* gene cause holoprosencephaly (HPE), suggesting an important role for TGIF function in embryogenesis [24, 25]. In mice, loss of Tgif1 function does not have severe phenotypic consequences, at least in a mixed strain background [19, 26–28]. Similarly, *Tgif2* null mice are normal on a mixed strain background. The combination of both mutations results in early embryonic lethality, with gastrulation defects, in all embryos that are homozygous null for both genes [29]. In the background of a *Tgif2* null mutation, conditional *Tgif1* deletion using Sox2Cre, which is expressed throughout the embryo proper from around day 5.5 of gestation, allows double null embryos to progress beyond gastrulation. However, these conditional double null embryos have HPE and left-right asymmetry defects and the majority do not survive past embryonic day 11 [29, 30]. While this suggests an essential role for TGIF function early in embryogenesis, the function of Tgif1 and Tgif2 later in development is less well understood. Although Tgif1 and Tgif2 single null mice are relatively normal on a mixed strain background, transferring a *Tgif1* mutation to a relatively pure C57BL/6 strain results in approximately 50% perinatal lethality of the *Tgif1* nulls, placental defects and otitis media in the weaned mice [31, 32]. Thus, the C57BL/6 background may be permissive for uncovering strain-specific phenotypes associated with Tgif function.

Here we show that on a relatively pure C57BL/6 strain background, about half the *Tgif1* null embryos have severe defects in rib and vertebra patterning, primarily affecting the lower cervical and upper thoracic region. In adult mice lacking Tgif1 we found similar defects in the cervical vertebrae, but no rib defects. The combination of mutations in both *Tgif1* and *Tgif2* resulted in more severe skeletal defects, although mutation of *Tgif2* alone had a relatively minimal effect. Finally, we show that *Tgif1* mutant embryos that are exposed to RA *in utero* have more frequent rib defects.

## Results

### Cervical vertebra defects in *Tgif1* null mice

**Fig 1:**
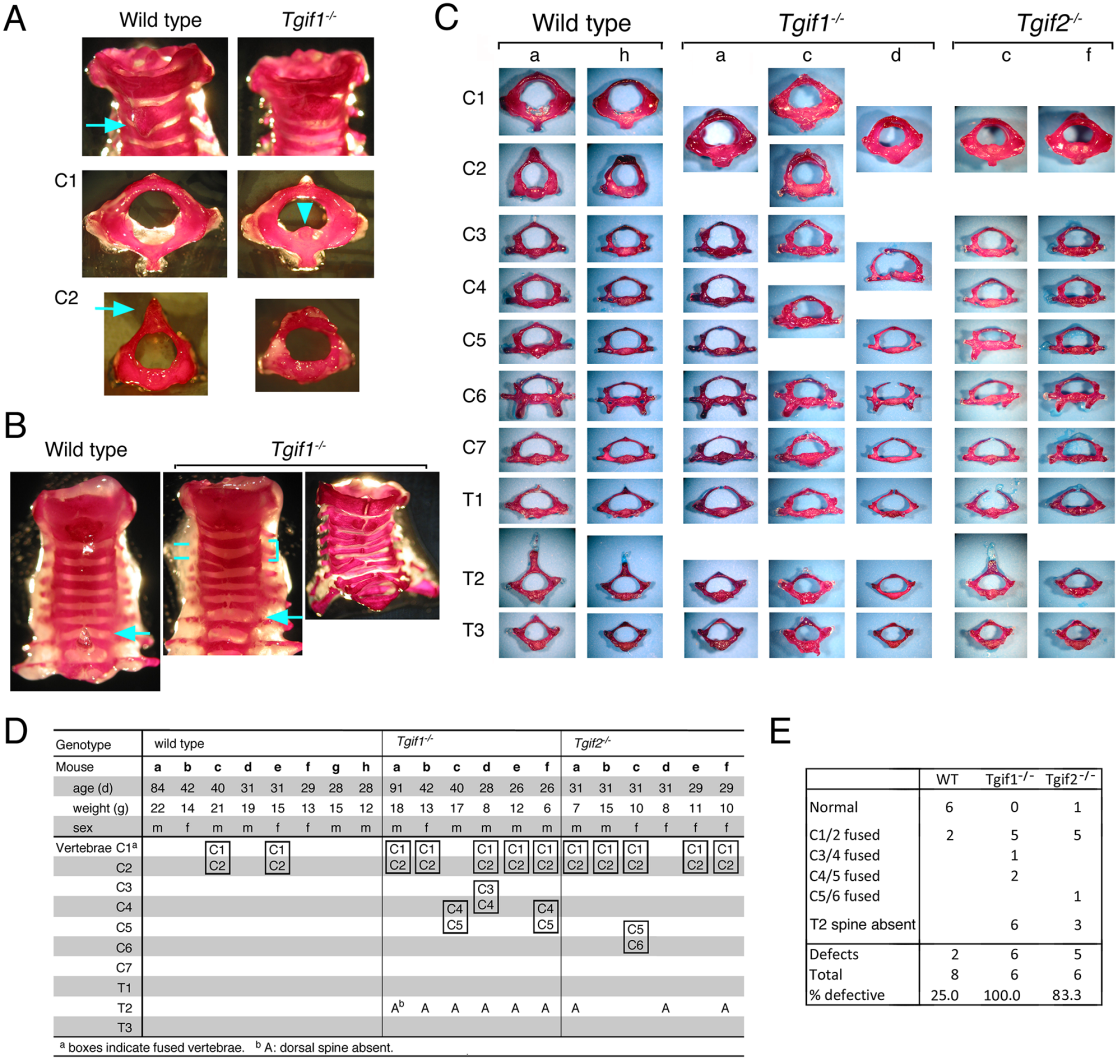
Vertebra defects in adult mice. A, B) Alizarin red stained cervical vertebrae from wild type and *Tgif1* null mice are shown. Note the absence of the dorsal spine on C2 in the *Tgif1* null (A, arrowed in wild type), and the ectopic bone in C1 (arrowhead in A) in the *Tgif1* null. In B, the partially fused C4 and C5 are indicated by a bracket, and T2 is indicated by an arrow. C) Separated alizarin red stained vertebrae, from C1 to T3 are shown from representative mice. The phenotypes of the mice shown in C and S1 Fig are listed in panel D, and summarized in panel E.

Although *Tgif1* null mice are quite normal on a mixed strain background, on a relatively pure C57BL/6 background around 50% of the *Tgif1* null mice die perinatally [31]. Further analysis of *Tgif1* null mice and late embryos in the C57BL/6 background revealed the presence of defects in skeletal patterning. In adult *Tgif1* null mice we observed frequent defects in cervical and upper thoracic vertebrae. In the majority of the *Tgif1* null animals examined, the C1 (atlas) and C2 (axis) vertebrae were fused, or very tightly interlocked. Comparison of the mutant atlas with the control revealed that the canal for the odontoid peg was filled with bone (Fig 1A). The axis in the mutant was generally malformed and lacked the dorsal neural spine. All of the *Tgif1* null mice had some degree of C1 and C2 malformation, although the severity varied. Further comparison of the cervical vertebrae revealed additional defects in the *Tgif1* nulls (for example, see Fig 1B). The severity of the defects in the lower cervical vertebrae was quite variable, and included what appeared to be split or fused vertebrae, primarily affecting C3-C5. Closer examination suggested that in most cases one half of a vertebra was missing, or fused to the adjacent one (Fig 1B). Thus on one side the two vertebrae were fused into a single bone, but on the other side there appeared to be two separate vertebrae, at least on the dorsal side. These defects in C3-C5 affected about half of the *Tgif1* null animals (Fig 1C, 1D and S1 Fig). All *Tgif1* null animals examined also lacked the pronounced dorsal neural spine on the T2 vertebra (Fig 1). Since Tgif1 and Tgif2 share overlapping function during embryogenesis, we examined *Tgif2* null mice on a similar C57BL/6 background for vertebra defects. At embryonic day 18.5 (E18.5), *Tgif2* null embryos were present at close to the expected frequency in heterozygous intercrosses, whereas by weaning at P21, significantly fewer than expected *Tgif2* null mice were obtained, suggesting perinatal lethality as seen with *Tgif1* nulls in the C57BL/6 background (data not shown). Although *Tgif2* nulls were present at the right frequency at E18.5 they were generally smaller than wild type or heterozygous littermates in this background. Thus both *Tgif1* and *Tgif2* null mutations in the C57BL/6 background result in reduced embryo growth and perinatal lethality. We examined several *Tgif2* null mice at around 4–5 weeks after birth for cervical vertebra defects. The majority had fused C1-C2 vertebra as seen in *Tgif1* nulls, and half lacked the T2 dorsal spine, but we did not observe severe defects in the C3-C5 vertebrae, although one had a partial fusion of C5 and C6 (Fig 1C–1E and S1 Fig). These data suggest that loss of *Tgif1*, and to a lesser degree *Tgif2*, may affect vertebral development, primarily affecting fusions of the cervical vertebrae.

### Rib and vertebra defects in *Tgif1* null embryos

**Fig 2:**
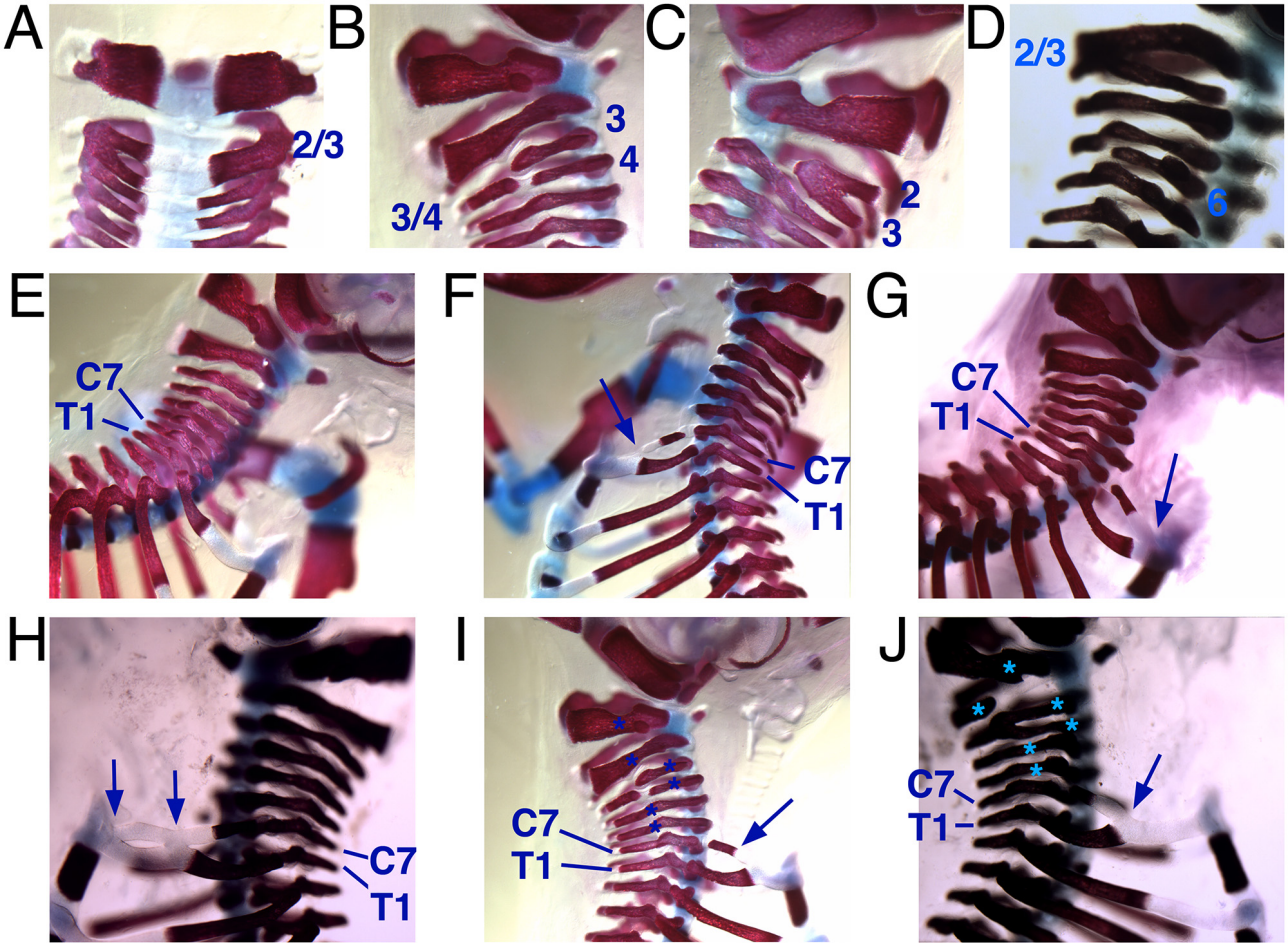
Rib and vertebra defects in *Tgif1* mutant E18.5 embryos. E18.5 embryos stained with alizarin red and alcian blue are shown focused on the cervical vertebrae or upper thoracic region. A-D) *Tgif1* mutant embryos with defects in cervical vertebra are shown. Affected vertebrae are indicated with numbers, where 2/3 and 3/4 represent fused vertebrae. E) A wild type embryo with the anterior most rib on T1. F, G) *Tgif1* mutant embryos with ectopic ribs on C7 (arrows), which join either the lower rib (F) or the sternum (G). H) A *Tgif1* mutant embryos with an ectopic C7 rib that joins both sternum and the lower rib at the junction between bone and cartilage (arrows). I, J) Examples of *Tgif1* null embryos with posterior transformation (arrows) and cervical vertebra defects (stars). C7 and T1 vertebrae are indicated in panels E-J. The same embryo is shown in panels B and I.

**Fig 3:**
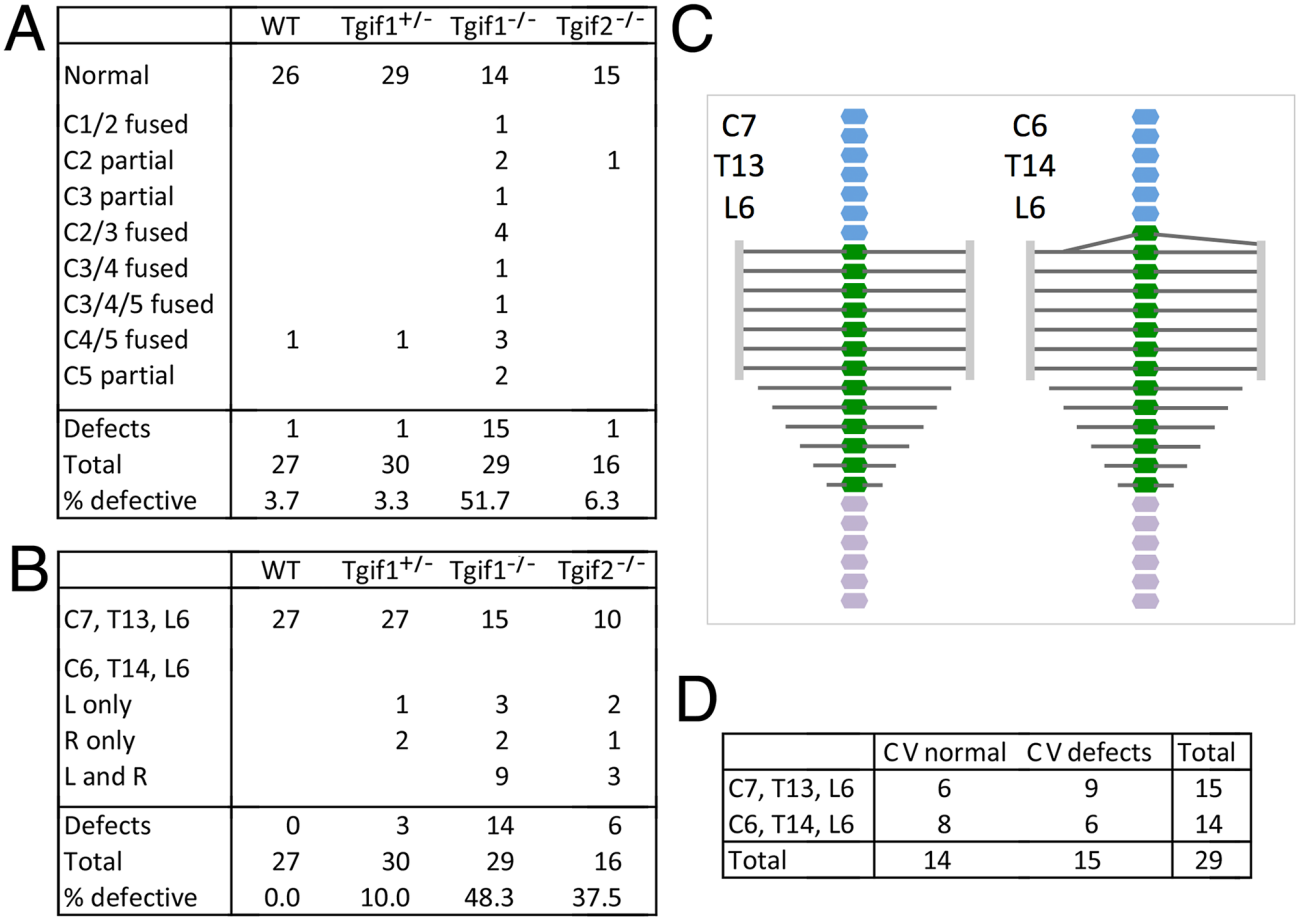
Rib defects in *Tgif1* and *Tgif2* mutants. A) The number (and percentage) of embryos of each genotype with defects the cervical vertebrae are summarized. Defects observed were fusion of two adjacent vertebrae (fused, in figure), or loss of one vertebra on one side of the embryo (partial, in figure). B) Axial patterning defects are summarized for each genotype. Embryos with C6, T14, L6 are separated out depending on whether this posterior transformation was seen on both sides or on left (L) or right (R) only. The percentage with any defect is shown. C) The normal (C7, T13, L6) and defective (C6, T14, L6) patterns are shown schematically. D) Co-occurrence of the posterior transformation phenotype and defects in the cervical vertebrae is summarized for *Tgif1* null embryos.

To further examine the effects of loss of *Tgif1* on skeletal development, we analyzed embryos at E18.5 from *Tgif1* heterozygous intercrosses. Examination of the cervical vertebrae revealed similar defects to those seen in adult mice, primarily affecting the C2-C5 vertebrae (Fig 2A–2D). These cervical vertebra defects affected about half of the *Tgif1* null embryos, but were rarely seen in heterozygotes or wild-type littermates (Fig 3A). While examining the cervical vertebrae we also noticed that many of the *Tgif1* null embryos had ribs extending from the C7 vertebra. In wild-types, and a proportion of the *Tgif1* nulls, the first rib is present on the T1 vertebra (Fig 2E). In many wild type embryos a small piece of bone was visible adjacent to the C7 vertebra on one or both sides, but this rib anlagen had not extended to form an elongated rib-like structure, a phenotype that has been documented previously in wild type mice [33]. In contrast, in a proportion of the *Tgif1* null embryos a full rib was present that either connected C7 to the T1 rib, or extended all the way to the sternum (Fig 2F and 2G). We also observed C7 ribs that were connected both to the T1 rib and the sternum (Fig 2H). If the C7 rib joined the T1 rib it usually did so at the boundary between the bone and cartilage. The ribs on C7 generally affected both left and right sides of the embryo, and were found in almost half the *Tgif1* null E18.5 embryos examined (Fig 3B). Even when there was a full rib on one side only, this did not affect the overall register of the sternum, and we observed a crankshaft sternum phenotype only rarely (in one embryo of each genotype). We did not observe any changes in the lower thoracic or lumbar regions, such that this represented an additional rib. Assuming the ectopic C7 rib is indicative of a posterior transformation of the C7 vertebra, these defects resulted in a change in the axial formula from the normal 7 cervical, 13 thoracic (with ribs), 6 lumbar (C7, T13, L6) to C6, T14, L6 (Fig 3C). This defect was not found in wild types and in only 10% of the *Tgif1* heterozygotes (Fig 3B). There was some overlap between the presence of cervical vertebra defects and extra ribs on C7 (for example, see Fig 2I and 2J), but the majority of affected *Tgif1* null embryos had one defect or the other (8 and 9 embryos with either defect versus 6 with both; Fig 3D). Examination of *Tgif2* null E18.5 day embryos revealed a slightly lower frequency of ectopic ribs on C7 and rare defects in the cervical vertebrae, consistent with the lower frequency in weaned mice (Fig 3A and 3B). Other skeletal patterning defects were not observed in *Tgif1* or *Tgif2* null embryos. Thus it appears that in addition to the cervical vertebra fusions seen in both adult mice and embryos, there is a high frequency of posterior transformation of C7 in *Tgif1* null embryos.

### Altered *Hoxc6* expression in *Tgif1* null embryos

**Fig 4:**
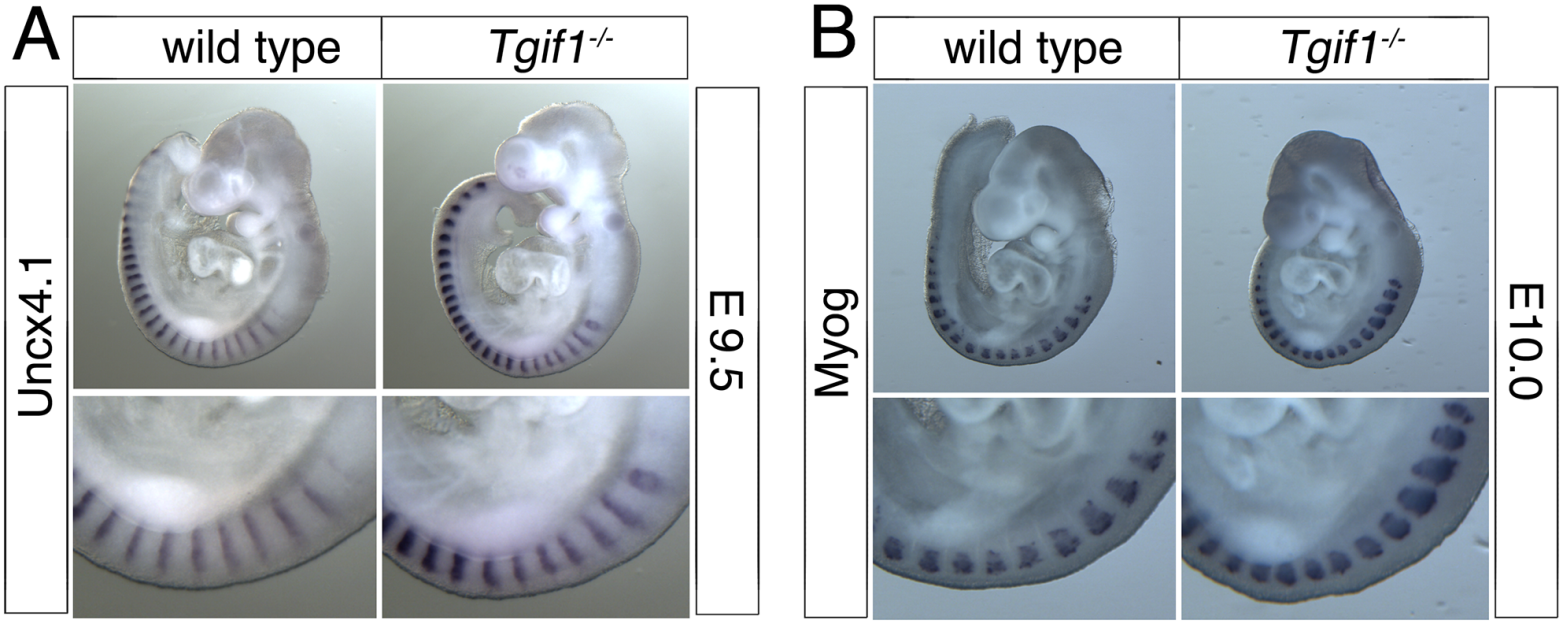
Normal somites in *Tgif1* null embryos. Wild type and *Tgif1* null embryos at E9.5-E10.0 (as indicated) were analyzed by whole-mount *in situ* hybridization, for *Uncx4*.*1* (A) and *Myogenin* (B). A higher resolution image of part of the embryos is shown below.

**Fig 5:**
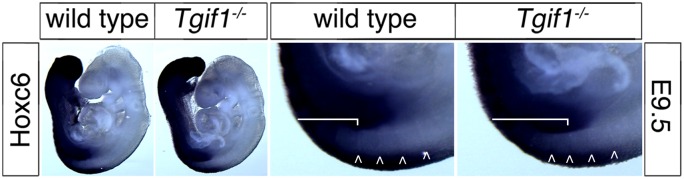
Analysis of *Hoxc6* expression. Wild type and *Tgif1* null embryos at E9.5 were analyzed by whole-mount *in situ* hybridization, for *Hoxc6*. A higher resolution image of part of the embryos is shown to the right. The bracket indicates the extent of strong *Hoxc6* staining in the somites, and the arrows indicate somite boundaries.

**Fig 6:**
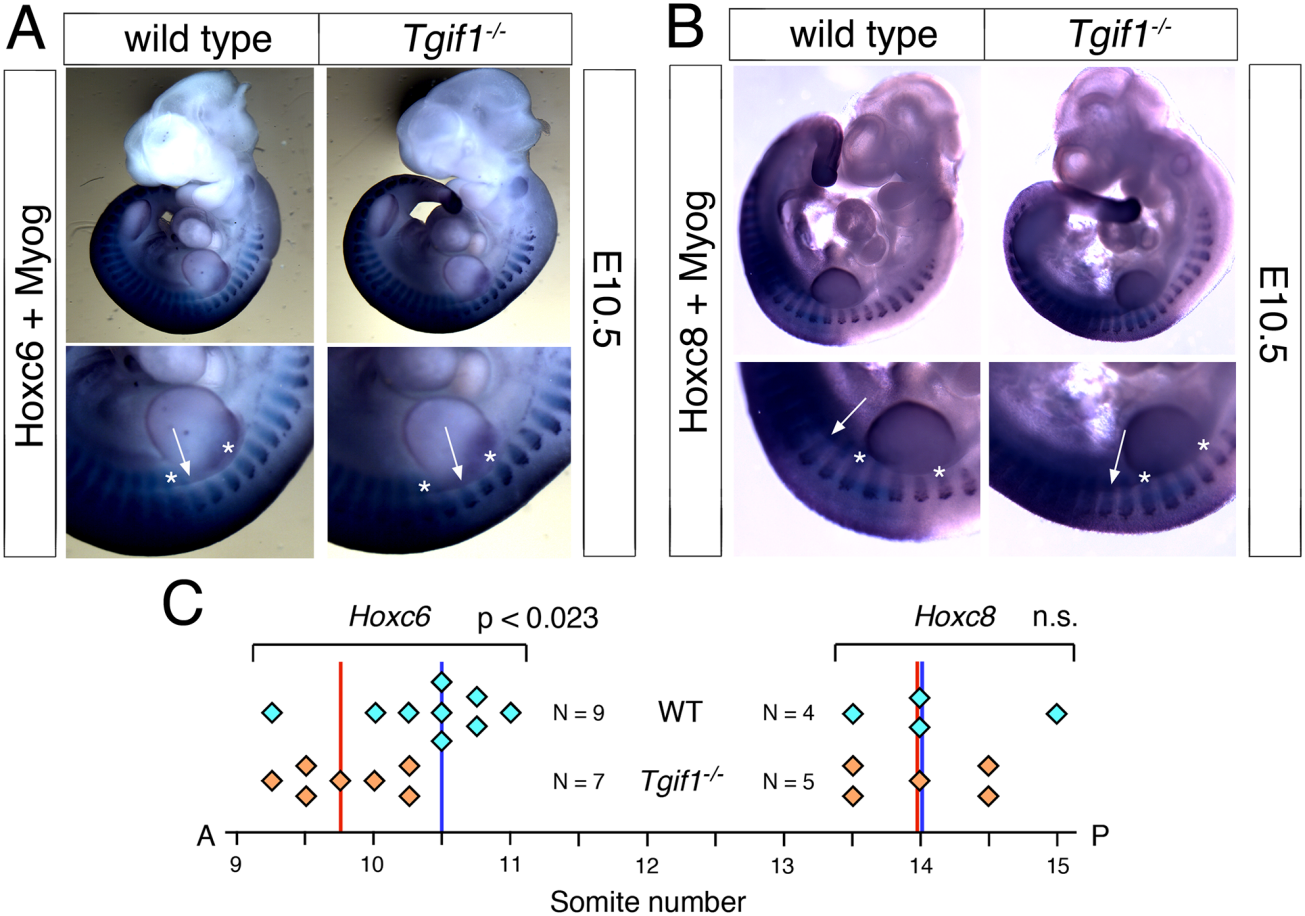
Altered *Hoxc6* expression in *Tgif1* null embryos. Wild type and *Tgif1* null embryos at E10.5 were analyzed by whole-mount *in situ* hybridization, for *Hoxc6* and *Myogenin* together (A) and for *Hoxc8* and *Myogenin* (B). A higher resolution image of part of the embryos is shown below. The stripes of *Myogenin* expression in somites 8 and 12 are indicated by stars, and the approximate anterior boundaries of *Hoxc6* and *Hoxc8* expression are shown by arrows. C) The anterior boundaries of *Hoxc6* and *Hoxc8* expression in all embryos analyzed are shown schematically, together with the median boundaries (vertical lines: red—*Tgif1* null, blue—wild-type).

**Fig 7:**
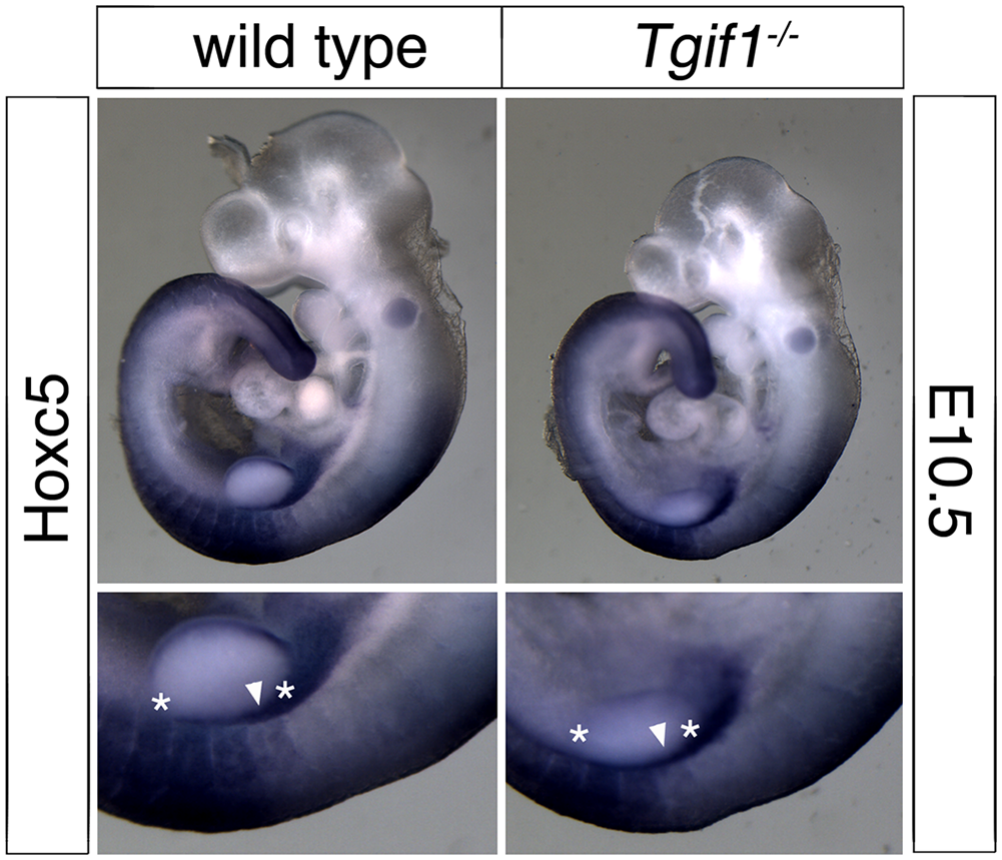
Analysis of *Hoxc5* expression. Wild type and *Tgif1* null embryos at E10.5 were analyzed by whole-mount *in situ* hybridization, for *Hoxc5*. Images of representative embryos are shown, with a higher resolution image of part of the embryos below. Two of four wild type embryos had this expression pattern, with the other two having slightly higher staining in somite 9 on one side of the embryo. Of the five *Tgif1* null embryos, three had the pattern shown and the remaining two had slightly weaker expression in somite 9 on one side of the embryo. Somites 8 and 12 are indicated by stars, and the arrowhead shows the expression in somite 9.

The expression of *Hox* genes is known to regulate patterning of the vertebrae and ribs, and changes in the anterior boundaries of *Hox* gene expression can result in patterning defects in the antero-posterior axis [34]. We were, therefore, interested to know whether *Hox* gene expression was affected in *Tgif1* null embryos. To examine whether somites developed normally in *Tgif1* null embryos, we first tested *Uncx4*.*1*, which is expressed in a single stripe in each somite, by whole mount *in situ* hybridization (WISH). As shown in Fig 4A, *Uncx4*.*1* expression was similar between wild type and *Tgif1* null embryos at E9.5, suggesting that somitogenesis has proceeded normally. Similarly, *Myogenin* expression was observed in the expected pattern in *Tgif1* null embryos, with one band of expression per somite (Fig 4B). Since the defects we observed appeared to center on the boundary between the cervical and thoracic vertebrae, we next performed WISH for *Hoxc6*, which is expressed in the posterior of the embryo with an anterior boundary around somite 10–12, which corresponds to the C7-T1 vertebrae [34, 35]. Using a probe to *Hoxc6*, it appeared that there was a small anterior shift in the expression in the somites in *Tgif1* null embryos at E9.5 (Fig 5). To determine the anterior boundary of *Hoxc6* expression more precisely, we analyzed embryos with both *Myogenin* and *Hoxc6* probes together, allowing us to use the *Myogenin* expression pattern as a positional marker. As shown in Fig 6A, strong *Hoxc6* expression was present up to somites 9–10 in the mutant embryo, whereas in the wild type expression extended anterior to somites 10–11. Since the position of the anterior boundary of *Hoxc6* expression is somewhat subjective, 16 embryos (9 wild type and 7 *Tgif1* mutant) stained for *Myogenin* and *Hoxc6* were scored blind for the anterior *Hoxc6* boundary. The embryos shown in Fig 6A are representative of the differences observed. The median expression boundary for wild type was somite 10.5, and for *Tgif1* null was somite 9.75. Eight of the nine wild type boundaries fell between somites 10 and 11 (Fig 6C), and all seven of the mutants between 9.25 and 10.25 (the apparent anterior boundary was scored to the nearest half somite on both sides, and an average taken for each embryo). Since the patterning defects centered on the C7-T1 region we also examined *Hoxc8* expression as a comparison, to determine whether there was a more general shift in Hox expression patterns, or whether it was specific to the affected region. Comparison of four wild-type and five *Tgif1* null embryos revealed a median anterior expression boundary of somite 14 in both, suggesting that the shift in anterior expression does not affect all regions of the embryo (Fig 6B and 6C). To test whether there was any change in expression of a Hox paralog that normally has a more anterior expression boundary than *Hoxc6*, we examined expression of *Hoxc5*. In embryos hybridized with a *Hoxc5* probe somite boundaries were still visible, so we compared expression between wild type and *Tgif1* null embryos hybridized to *Hoxc5* alone (Fig 7). Of the four wild type and five *Tgif1* null embryos analyzed all showed clear staining in somite 10 with a more variable signal in somite 9, but without any anterior expansion of expression in the *Tgif1* nulls. Thus there appears to be a relatively specific shift in the anterior boundary of *Hoxc6* expression in *Tgif1* null embryos, consistent with the observed posterior transformation of C7.

### Increased severity of skeletal defects in *Tgif1*;*Tgif2* double mutants

**Fig 8:**
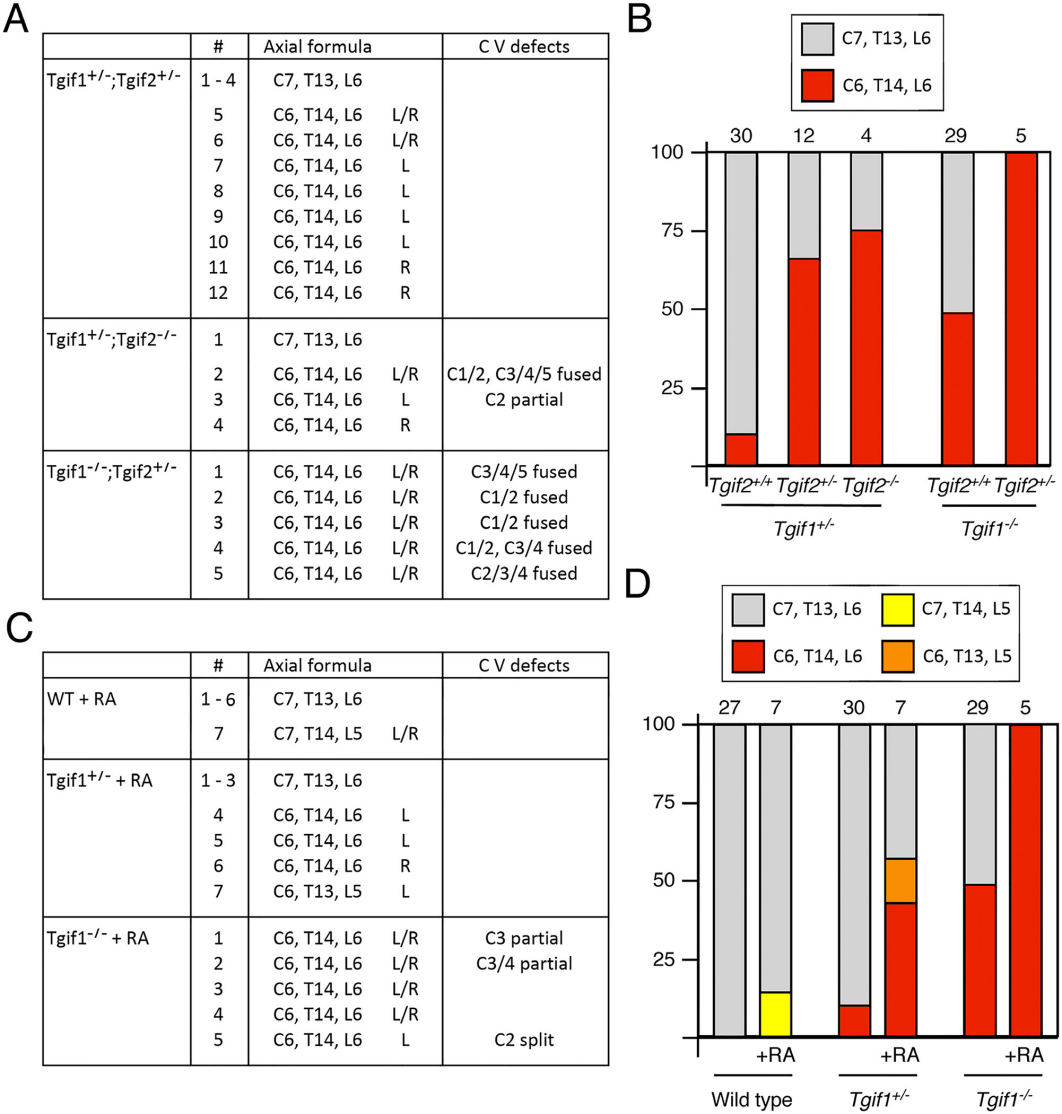
Combination of *Tgif1* and *Tgif2* mutations. A) The axial formulas and the presence of cervical vertebra defects is shown for E18.5 embryos from *Tgif1;Tgif2* double heterozygous intercrosses. # = the embryo number. For example a total of 12 double heterozygous embryos were analyzed, with embryos 1 to 4 being normal. The phenotypes for embryos 5 to 12 are listed individually. B) The percentage of embryos of the indicated genotypes with either normal or posterior transformation is shown, based on the embryos detailed in 3B and 8A. C) Data for E18.5 embryos from *Tgif1* heterozygous intercrosses, in which the embryos were exposed to 10mg/kg retinoic acid (ATRA) at E8.5. D) the percentage of embryos with different axial formulas are shown for embryos from *Tgif1* intercrosses (from 3B) compared to those treated with ATRA.

We have previously shown that the combination of homozygous mutations in both *Tgif1* and *Tgif2* results in synthetic phenotypes, including embryonic lethality and HPE, suggesting overlapping function, at least during early embryogenesis [29, 30]. Given the presence of skeletal defects in both *Tgif1* and *Tgif2* null embryos, we were interested to know whether combining mutations in both genes in the C57BL/6J background would have a more severe effect on rib and vertebra patterning. *Tgif1;Tgif2* double heterozygous mice were generally normal and fertile in the C57BL/6J background. Very few mice with homozygous null mutations in one gene and a heterozygous mutation in the other were weaned from double heterozygous intercrosses. Additionally, at E18.5, embryos with only one wild-type *Tgif1* or *Tgif2* allele were significantly smaller than their littermates (data not shown). However, at E18.5 we were able to isolate a small number of embryos that were not severely developmentally delayed and had three mutant alleles. We analyzed these embryos, as well as 12 double heterozygotes, for rib and vertebra defects. The addition of a heterozygous mutation in *Tgif2* to a heterozygous *Tgif1* mutation, resulted in an increase in the frequency the posterior transformation of the C7 vertebra, but did not increase cervical vertebral fusions (Fig 8A and 8B). The combination of a homozygous *Tgif2* mutation with the *Tgif1* heterozygous also increased the frequency of C7 transformations and some defects in the upper cervical vertebrae were also present. Of the five *Tgif1* null embryos that also had a heterozygous *Tgif2* mutation, all had bilateral ectopic ribs on C7 and variable defects in C1-C5, further supporting synergy between the two mutations (Fig 8A and 8B). Thus mutations in *Tgif2* combined with a *Tgif1* mutation, result in an increased frequency of the more severe defects seen *Tgif1* null embryos.

### *Tgif1* mutant embryos are sensitized to retinoic acid induced skeletal defects

Exposure of embryos to RA has been shown to alter the expression boundaries of many *Hox* genes, and to cause patterning defects that affect rib and vertebra specification [34]. Embryos treated at E7.0–7.5 had posterior transformations including ectopic ribs on C7. We have shown that exposure of *Tgif1* mutant embryos in a mixed strain background to RA at E7.5 increased the frequency of exencephaly at E13.5, suggesting a sensitization to RA-induced teratogenesis in *Tgif1* mutants [19]. To test whether embryos lacking *Tgif1* were more sensitive to RA-induced rib and vertebra patterning defects, we performed *Tgif1* heterozygous intercrosses, treated the pregnant females with RA at E7.5 or E8.5, and examined embryos at E18.5. In this relatively pure strain background, we recovered very few embryos from animals treated on day 7.5, and none of the embryos were *Tgif1* null. From treatments at E8.5, we did obtain a small number of informative litters at E18.5. At this dose at E8.5, we did not observe any defective cervical vertebrae or vertebro-sternal ribs in wild type embryos, although one of the seven wild types had an anterior transformation of L1, resulting in an axial formula of C7, T14, L5 (Fig 8C). Four out of seven of the *Tgif1* heterozygotes had an ectopic rib on C7 on one side, and all of the *Tgif1* homozygous null embryos that had been exposed to RA *in utero* had C7 ribs, with four of five having C7 ribs on both left and right. Other than some upper cervical vertebral fusions seen in the *Tgif1* nulls, we did not observe other axial patterning defects except for the single C7, T14, L5 wild type embryo and a *Tgif1* heterozygote with only five lumbar vertebrae (Fig 8C and 8D). This suggests that the combination of RA exposure and decreased *Tgif1* function results in a more severe patterning defect than either alone. Taken together with the previous analysis, these results suggest that loss of Tgif1 causes patterning defects that primarily affect the cervical and upper thoracic vertebrae. The severity and frequency of these defects can be increased by mutations in *Tgif2* or by exposure to RA.

## Discussion

Loss of TGIF function has been shown to cause multiple defects during early embryogenesis, but less is known about later effects of Tgif1 and Tgif2 on development. Here we demonstrate that loss of *Tgif1* causes axial patterning defects that are enhanced by mutations in *Tgif2*.

We and others have shown that loss of Tgif1 function does not cause severe phenotypic consequences in mice of a mixed strain background [19, 26–28]. Similarly, *Tgif2* null mice are normal on a mixed strain background. However, the combination of both mutations results in gastrulation defects and embryonic lethality [29]. A conditional mutation of *Tgif1* in a *Tgif2* null background bypasses the gastrulation defects, and embryos survive to around E11, with HPE-like phenotypes and defects in left-right asymmetry [30]. This clearly suggests some overlap in function, at least during early embryogenesis. Transferring the *Tgif1* null mutation to a relatively pure C57BL/6 strain background resulted in lethality in about 50% of the *Tgif1* homozygous nulls, and placental defects that were particularly severe in homozygous null females [31]. Similar placental defects, as well as otitis media were found in a second line of *Tgif1* null mice in a pure C57BL/6 strain background [32]. A *Tgif1* mutation that removes part of the coding sequence, and could potentially result in the expression of a truncated Tgif1 polypeptide also appeared to cause strain specific developmental defects [36]. Thus it appears that the C57BL/6 strain background is permissive for uncovering defects associated with loss of TGIF function. *Tgif2* heterozygous intercrosses in a relatively pure C57BL/6 strain produced significantly fewer *Tgif2* null mice at weaning than would be expected, and at E18.5, *Tgif2* null embryos were on average smaller than their littermates, consistent with strain-specific defects in *Tgif2* null mice. The data presented here suggest that in the C57BL/6 strain background loss of Tgif1, and to a lesser degree, Tgif2 results in defects in axial skeleton patterning, that are not observed in a mixed strain. As with the effects on early embryogenesis in a mixed strain, there is a synthetic effect of combining mutations in both genes.

Axial patterning is determined in part by the appropriate spatial and temporal control of expression of specific Hox genes [37, 38]. Gain and loss of function mutations in many Hox genes result in posterior or anterior transformations of the prevertebrae at specific positions along the axis. The defects observed in the absence of TGIF function appear to center on posterior transformation of the C7 vertebra, with some additional effects that result in fusions of the upper cervical vertebrae. Hox paralogs are expressed at different positions along the embryonic axis, with defined anterior boundaries. The anterior expression boundary of Hox group 6 paralogs is around somite 10–12, which corresponds to vertebrae C6 to T1 [34, 35]. Embryos with homozygous loss of function alleles in *Hoxa6*, *Hoxb6* and *Hoxc6* lack ribs on the T1 vertebra [39]. In contrast, over-expression of *Hoxb6* in the presomitic mesoderm results in ectopic ribs on all cervical vertebrae, suggesting that increased Hox activity can cause posterior transformations that convert cervical to thoracic vertebrae [40]. Here we show that in the absence of Tgif1, *Hoxc6* expression is altered, with an apparent shift in the anterior expression boundary of about one somite. In contrast, we did not observe any significant expansion of the *Hoxc8* or *Hoxc5* expression domains, suggesting that the effect of *Tgif1* mutation is relatively specific. The lack of an anterior expansion of the *Hoxc5* expression domain in *Tgif1* mutants perhaps suggests that the cervical vertebral fusions observed in these embryos are not due to homeotic transformations. One possibility is that small defects in segmentation in the Tgif1 mutants might contribute to vertebral fusions, although we do not have any evidence for this. However, the additional ribs on C7, together with the anterior expansion of *Hoxc6* expression likely indicate a posterior transformation phenotype at the cervical/thoracic boundary.

An *in vivo* role for TGIF function in regulating TGFβ family signaling is supported by analysis of *Tgif1;Tgif2* double null embryos, which have severe gastrulation defects, even in a mixed strain [29]. These defects, as well as changes in gene expression can be partially rescued by reducing the dose of Nodal, which signals to Smad2. Similarly, a partial rescue of left-right asymmetry defects and HPE-like phenotypes by *Nodal* heterozygosity was seen in *Tgif1;Tgif2* double null embryos created using a conditional allele of *Tgif1* and a *Sox2-Cre* transgene that deletes in the embryo proper, but not in extra-embryonic tissue [29, 30]. These results clearly place TGIFs within the Nodal/Smad signaling pathway and suggest that they inhibit gene expression activated through this pathway. Mutations in the genes encoding Activin receptor type IIA and type IIB have been shown to affect axial patterning, with defects in the number of thoracic vertebrae and vertebro-sternal ribs [41, 42]. ActRIIA and ActRIIB (encoded by *Acvr2a* and *Acvr2b*) mediate signaling from the TGFβ family member, Gdf11, to Smad2 [42]. Embryos that lack ActRIIB have a posterior shift in the anterior expression boundary of *Hoxc6*, *c8*, *c9* and *c10*, and have patterning defects primarily in the caudal thoracic vertebrae. Most *Acvr2b* null embryos have an increase in the number of thoracic vertebrae and in the number of vertebro-sternal ribs, but still have the first (anterior-most) rib on T1, with the appropriate number of cervical vertebrae [41]. This phenotype is exacerbated in *Acvr2b* nulls with a heterozygous mutation in *Acvr2a*, again without affecting the cervical vertebrae [42]. Although we have not combined a *Tgif1* mutation with Activin receptor mutations, our other attempts to test whether the effects of Tgif1 loss of function causes axial defects via TGFß family signaling suggested that this phenotype may be independent of TGFß family signaling (data not shown).

Treatment of embryos with RA causes a number of axial patterning defects, dependent in part on the timing of the treatment [34]. Treatment at E7-7.5 resulted in posterior transformation of the C7 vertebrae (primarily resulting in C6, T13, L6 or C6, T14, L5), whereas at E8.5, the major phenotype was anterior transformation at the thoracic-lumbar boundary, giving C7, T14, L6 [34]. In the strain background used here (C57BL/6 and heterozygous for *Tgif1*) we observed some similar effects of RA treatment on axial patterning. Treatment at E7.5 resulted in too few viable embryos by E18.5, of which none were *Tgif1* null, suggesting that this strain may be very sensitive to RA-mediated teratogenesis. In this context, we have already shown that *Tgif1* mutations in a mixed strain background resulted in increased RA-induced developmental defects [19]. When we treated with RA at E8.5, two embryos had anterior transformations at the thoracic-lumbar boundary, consistent with previous work. As with the addition of a *Tgif2* mutation, *in utero* exposure of *Tgif1* heterozygous and homozygous null embryos to RA at E8.5, resulted in an increase in the severity and penetrance of the posterior transformation phenotype, primarily without changing the type of axial defects observed. It is possible that with more embryos, or different timing, other synthetic effects of RA and *Tgif* mutation might be uncovered. However, from this analysis it seems that the major effect of RA on *Tgif1*-mutant phenotypes centers on the cervical/thoracic boundary. Exposure of *Acvr2b* mutants to RA at E8.5 also increased the severity of the defects, but unlike in our *Tgif1* mutants, cervical vertebra defects were not observed at a high frequency [41]. Together, these analyses suggest that *in utero* exposure to RA can on its own cause axial patterning defects, and that it can also increase the severity or penetrance of defects caused by alterations in other signaling pathways.

Tgif1 has been suggested to regulate several signaling pathways, including TGFß, retinoic acid and more recently, Wnt signaling [13, 19, 43]. In addition, recent ChIP-seq analysis in mouse ES cells has revealed a large number of potential Tgif1-associated regions across the genome, many of which have matches to the consensus TGIF binding site [23]. It is, therefore, difficult at present to speculate on the exact mechanism by which loss of Tgif1 causes the defects described here. Signaling by GDF11 via Activin receptors appears to primarily affect axial patterning in the thoracic/lumbar regions, although there is a posterior shift in *Hoxc6* expression in embryos lacking AcRIIB [42]. Thus it is possible that in the absence of Tgif1 increased signaling via ActRIIB would result in an anterior *Hoxc6* shift. However, our attempts to link the *Tgif1* mutant phenotypes to signaling via TGFß/Activin receptors have not been successful. One alternative is that increased RA signaling in the absence of Tgif1 causes the posterior transformation at C7, although excess RA causes multiple defects in axial patterning, and the synthetic effects of *Tgif1* mutation and RA treatment are certainly also consistent with effects in two independent pathways. Finally, given the potentially large number of Tgif1 binding sites across the genome it is tempting to speculate that the phenotypes observed here are caused by Tgif1 transcriptional effects that are independent of both TGFß and RA.

In summary, we show that Tgifs control axial patterning, and appear to function at a relatively specific position in the axis, with defects centered on the cervical-thoracic boundary. Tgifs may fine-tune the activity of the pathways that define axial position, however, the direct mechanism of Tgif function in this process remains to be determined.

## Experimental Procedures

### Mice

*Tgif1* and *Tgif2* null mice have been described previously [19, 29, 31], and were maintained on a relatively pure C57BL/6 strain background, generated by six sequential back-crosses to C57BL/6 mice, starting with C57BL/6 X 129Sv mixed strain animals. All procedures were approved by the Animal Care and Use Committee of the University of Virginia. Mice were housed on a standard 12/12 light/dark cycle, fed ad libitum, and euthanized by CO_2_. Genomic DNA for genotype analysis was purified from ear punch (at P21), yolk sac (at E10.5), or from tail (at E18.5) by HotShot [44]. *Tgif1* heterozygotes were intercrossed and pregnant females were treated by gavage, at E8.5 with 10 mg/Kg all trans retinoic acid (Sigma) in 200μl corn oil.

### *In situ* hybridization

Whole-mount *in situ* hybridization was performed on 9.0–10.5dpc embryos per genotype with digoxigenin labeled riboprobes, as described [45]. Images of E10.5 embryos were captured using a Leica MZ16 stereomicroscope and QImaging 5.0 RTV digital camera. All images are representative of at least three embryos analyzed, except where noted in the legends.

### Alizarin red/alcian blue staining

Bone and cartilage staining was performed as described in [46]. Briefly, the skin and internal organs were removed from E18.5 embryos prior to ethanol fixation, followed by staining with Alcian Blue (Sigma), additional ethanol fixation, staining with Alizarin Red (Sigma), and clearing in potassium hydroxide solution. Adult skeletons were fixed in ethanol, then partially cleared in potassium hydroxide prior to staining with Alizarin Red. After staining, the adult bones were cleared further in potassium hydroxide solution prior to imaging. Images of bones were captured as for *in situ* analysis.

## Supporting Information

S1 Fig Analysis of cervical vertebrae in adult mice. Separated alizarin red stained vertebrae, from C1 to T3 are shown from wild type mice (W-a to W-h), or mice with homozygous null mutations in either Tgif1 (T1-a to T1-f), or Tgif2 (T2-a to T2-f). Skeletons were fixed in ethanol, then partially cleared in potassium hydroxide prior to staining with Alizarin Red. After staining, the bones were cleared further in potassium hydroxide solution prior to imaging. The phenotypes of these mice are summarized in Fig 1D and 1E.
(TIF)
