# Supplementary figures and images for: Tgif1 and Tgif2 Regulate Axial Patterning in Mouse
Source: PLoS One. 2016 May 17;11(5):e0155837. doi: 10.1371/journal.pone.0155837 (PMC4871493; doi:10.1371/journal.pone.0155837)

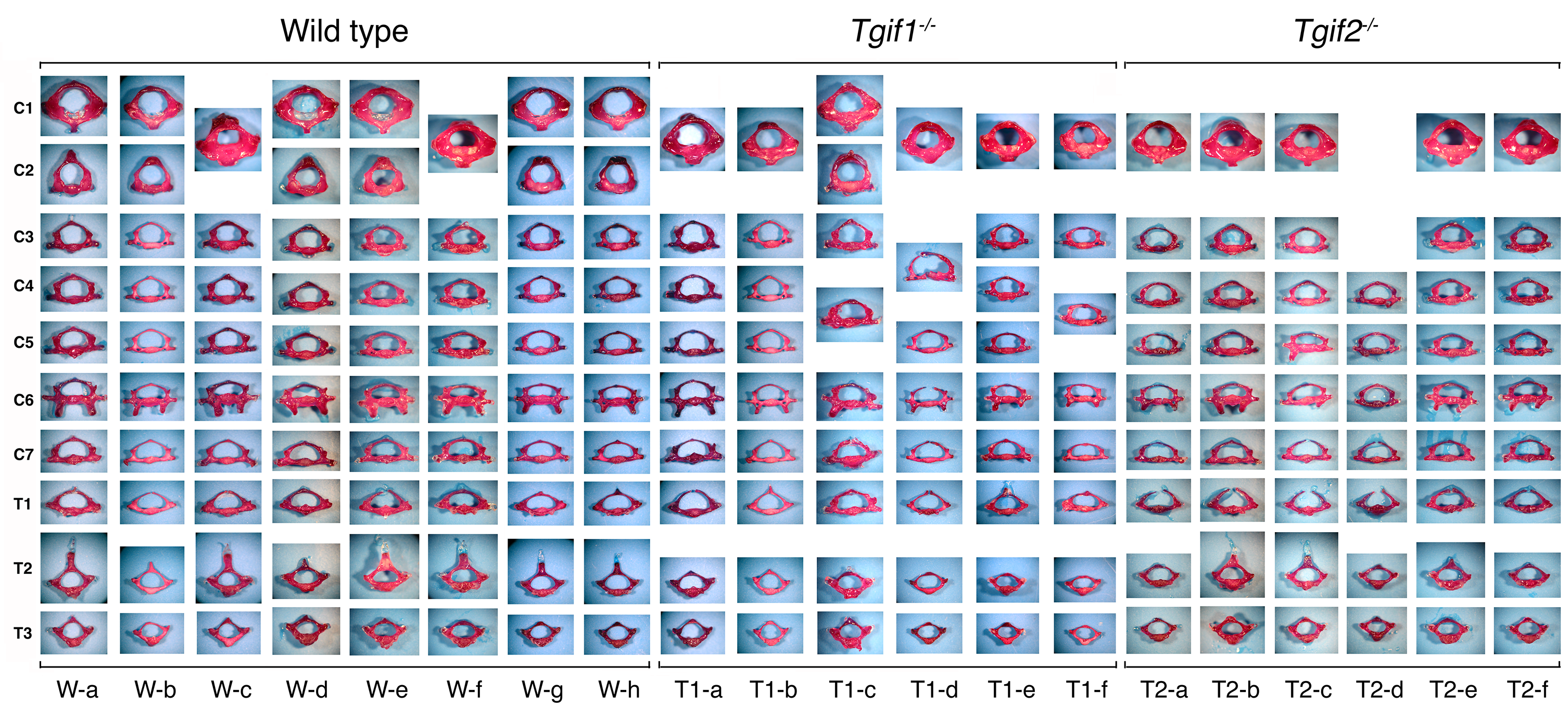

Supplement: S1 Fig — Separated alizarin red stained vertebrae, from C1 to T3 are shown from wild type mice (W-a to W-h), or mice with homozygous null mutations in either Tgif1 (T1-a to T1-f), or Tgif2 (T2-a to T2-f). Skeletons were fixed in ethanol, then partially cleared in potassium hydroxide prior to staining with Alizarin Red. After staining, the bones were cleared further in potassium hydroxide solution prior to imaging. The phenotypes of these mice are summarized in Fig 1D and 1E. (TIF) [file pone.0155837.s001.tif]
